# Prunetin Attenuates Dexamethasone‐Induced Pancreatic β‐Cell Apoptosis: Modulation of Oxidative Stress and MAPK Signaling but Not Phosphorylated C‐Abl Levels

**DOI:** 10.1002/cbf.70292

**Published:** 2026-08-24

**Authors:** Thaksaon Kittipassorn, Nawarat Rattanajearakul, Petcharee Maneethorn, Namoiy Semprasert, Suwattanee Kooptiwut

**Affiliations:** ^1^ Department of Physiology, Faculty of Medicine Siriraj Hospital Mahidol University Bangkok Thailand; ^2^ Laboratory of Alimentary Neuroscience, Department of Applied Life Sciences, Graduate School of Bioagricultural Sciences Nagoya University Nagoya Aichi Japan

**Keywords:** c‐Abl, dexamethasone, GSTP1, MAPK, oxidative stress, pancreatic β‐cell apoptosis, prunetin

## Abstract

Dexamethasone has been reported to induce pancreatic β‐cell apoptosis, which may contribute to steroid‐induced diabetes following long‐term glucocorticoid therapy. Although prunetin, a natural isoflavone, has been shown to attenuate dexamethasone‐induced pancreatic β‐cell apoptosis with suppression of p53 signaling as an associated pathway, the involvement of other signaling pathways remains unclear. This study aimed to investigate whether prunetin‐mediated attenuation of dexamethasone‐induced β‐cell apoptosis was associated with changes in phosphorylated c‐Abl (p‐c‐Abl) levels, oxidative stress, and MAPK signaling pathways. The rat insulinoma cell line INS‐1 was treated with dexamethasone in the presence or absence of prunetin, followed by Annexin V‐FITC/propidium iodide staining, western blotting, superoxide measurement, and caspase‐3/7 activity assays. Dexamethasone induced early apoptosis, reduced p‐c‐Abl and glutathione S‐transferase P1 (GSTP1) levels, increased superoxide generation, elevated phosphorylated JNK and p38 levels, decreased phosphorylated ERK1/2 levels, and stimulated caspase‐3/7 activity, whereas total c‐Abl levels remained unchanged. Prunetin pre‐treatment before dexamethasone administration significantly reduced early apoptosis, restored GSTP1 expression, attenuated oxidative stress, and modulated MAPK signaling by decreasing phosphorylated JNK and p38 levels while increasing phosphorylated ERK1/2 levels. However, prunetin did not restore the dexamethasone‐induced reduction in p‐c‐Abl levels. These findings suggest that prunetin attenuates β‐cell apoptosis induced by dexamethasone and that this effect is associated with modulation of oxidative stress, GSTP1 expression, and MAPK signaling pathways, but not with restoration of reduced p‐c‐Abl levels. Collectively, this study extends current knowledge of signaling changes associated with prunetin in glucocorticoid‐induced β‐cell injury.

## Significance statement

Long‐term glucocorticoid therapy can impair pancreatic β‐cells and contribute to steroid‐induced diabetes, yet the molecular events related to this injury remain incompletely understood. This study examines the natural compound prunetin and identifies signaling changes accompanying prunetin‐mediated attenuation of dexamethasone‐induced β‐cell apoptosis. The findings show that prunetin is associated with reduced oxidative stress, increased GSTP1 expression, and modulation of MAPK signaling, while not restoring dexamethasone‐suppressed p‐c‐Abl levels. By clarifying pathways linked to prunetin‐mediated mitigation of β‐cell apoptosis, this work expands current understanding of glucocorticoid‐induced β‐cell injury and provides a foundation for future studies exploring signaling targets relevant to steroid‐induced diabetes.

**Abbreviations**
**β**‐actin: beta‐actin
**β**‐cell: beta cell
°C: degrees celsius
c‐Abl: cellular Abelson tyrosine kinase
DMSO: dimethyl sulfoxide
DR5: TRAIL death receptor
ERK1/2: extracellular signal‐regulated kinase 1/2
FBS: fetal bovine serum
FITC: fluorescein isothiocyanate
GSK‐3β: glycogen synthase kinase‐3β
GSTP1: glutathione S‐transferase P1
JNK: c‐Jun N‐terminal kinase
KOH: potassium hydroxide
LPS: lipopolysaccharide
MAPK: mitogen‐activated protein kinase
NBT: nitro blue tetrazolium chloride
p38: p38 mitogen‐activated protein kinase
PBS: phosphate‐buffered saline
p‐c‐Abl: phosphorylated c‐Abl
p‐ERK1/2: phosphorylated ERK1/2
PI: propidium iodide
p‐JNK: phosphorylated JNK
p‐p38: phosphorylated p38
PVDF: polyvinylidene difluoride
RIPA: radioimmunoprecipitation assay
RPMI: Roswell Park Memorial Institute
TBST: Tris‐buffered saline with Tween‐20
TRAIL: tumor necrosis factor‐related apoptosis‐inducing ligand

## 1 Introduction

Glucocorticoids are widely prescribed to treat inflammatory and autoimmune diseases [1]. However, long‐term use of glucocorticoids, including dexamethasone, can lead to adverse effects, notably hyperglycemia and steroid‐induced diabetes [2, 3, 4, 5]. A meta‐analysis investigating steroid‐induced hyperglycemia and diabetes in non‐diabetic patients treated with glucocorticoids reported incidence rates of 32.3% for hyperglycemia and 18.6% for diabetes [6]. Steroid‐induced diabetes represents a substantial clinical burden on patients, their families, and society. Thus, a deeper understanding of its pathophysiology is essential and may facilitate the identification of potential therapeutic targets.

Dexamethasone contributes to steroid‐induced diabetes through multiple mechanisms, including increased hepatic glucose production, induction of insulin resistance, and promotion of pancreatic β‐cell apoptosis [7, 8, 9, 10]. Several pathways have been implicated in dexamethasone‐induced β‐cell apoptosis, such as upregulation of pro‐apoptotic proteins [10, 11], downregulation of anti‐apoptotic proteins [10, 11], activation of the p53 signaling pathway [11], activation of glycogen synthase kinase‐3β (GSK‐3β) [12], induction of tumor necrosis factor‐related apoptosis‐inducing ligand (TRAIL) and TRAIL death receptor (DR5) [13, 14], and increased oxidative stress [10]. Oxidative stress activates the cellular Abelson tyrosine kinase (c‐Abl), a non‐receptor tyrosine kinase implicated in cell cycle arrest and apoptosis [15, 16, 17, 18]. In β‐cells, c‐Abl expression and activation are induced by high glucose [19, 20]. High glucose also alters mitogen‐activated protein kinase (MAPK) signaling, particularly by enhancing phosphorylation of c‐Jun N‐terminal kinase (JNK) and p38 MAPK (p38) [21, 22, 23], stress‐activated kinases implicated in apoptotic signaling [24]. Given that dexamethasone promotes oxidative stress [10], which has been reported to activate c‐Abl and MAPK signaling, their activation may be associated with dexamethasone‐induced β‐cell apoptosis. However, whether dexamethasone activates c‐Abl in β‐cells remains unclear.

Prunetin is a natural O‐methylated isoflavone, a subclass of flavonoids, found in plants such as kudzu [25], red cherry [26], and licorice [27]. Prunetin has been reported to exert anti‐inflammatory [28], cytoprotective [11, 29], and antioxidant effects [29]. In liver tissues of streptozotocin‐induced diabetic nephrotoxic rats, prunetin reduced lipid peroxidation and enhanced activities of antioxidant enzymes, including superoxide dismutase, catalase, and glutathione peroxidase [30]. A previous study demonstrated that prunetin attenuated dexamethasone‐induced β‐cell apoptosis and p53 protein expression [11]. Nevertheless, the effects of prunetin on additional signaling pathways in β‐cells remain largely unexplored.

This study aimed to investigate whether dexamethasone altered phosphorylated c‐Abl (p‐c‐Abl) levels in pancreatic β‐cells and whether prunetin‐mediated attenuation of dexamethasone‐induced β‐cell apoptosis was associated with changes in p‐c‐Abl levels, oxidative stress, and MAPK signaling pathways.

## 2 Materials and Methods

### 2.1 Cell Culture

The male rat insulinoma cell line (INS‐1 832/13) (RRID:CVCL_7226) was purchased from Merck Life Science (Darmstadt, Germany). The cell line has not been reported as misidentified or contaminated in the International Cell Line Authentication Committee (ICLAC) database, the NCBI misidentified cell line database, or the Cellosaurus problematic cell line database. The cell line was free of *Mycoplasma* contamination as tested using a Mycoplasma PCR detection kit (abm, Richmond, BC, Canada). Cells were grown in RPMI 1640 medium with 2 mM l‐glutamine and 11.1 mM glucose without phenol red (Sigma‐Aldrich, St. Louis, MO, USA), containing 10% heat‐inactivated fetal bovine serum (FBS), 100 µg/mL streptomycin, and 100 U/mL penicillin. Cells were maintained at 37°C and 5% CO_2_ and passaged every 2 days.

### 2.2 Treatments

The treatments were dexamethasone (Sigma‐Aldrich, St. Louis, MO, USA) dissolved in phosphate‐buffered saline (PBS) and prunetin (Sigma‐Aldrich, St. Louis, MO, USA) dissolved in dimethyl sulfoxide (DMSO). INS‐1 cells were seeded and maintained for approximately 24 h. On the day of treatment, the medium was replaced with fresh medium containing the designated treatments. Four groups were established: (1) control, treated with DMSO; (2) prunetin alone, pre‐treated with prunetin (10 µM) for 1 h and then continuously incubated in the same medium; (3) dexamethasone alone, treated with dexamethasone (0.1 µM) without prunetin pre‐treatment; and (4) prunetin + dexamethasone, pre‐treated with prunetin (10 µM) for 1 h followed by dexamethasone (0.1 µM) without removing prunetin. Cells were treated for 48 h for western blotting and nitro blue tetrazolium chloride (NBT) reduction assay, and for 72 h for Annexin V‑fluorescein isothiocyanate/propidium iodide (Annexin V‐FITC/PI) assay and Caspase‐Glo 3/7 assay.

### 2.3 Annexin V‑Fluorescein Isothiocyanate/Propidium Iodide (Annexin V‐FITC/PI) Assay

To assess cell apoptosis, an Annexin V‐FITC/PI assay was performed using a FITC Annexin V Apoptosis Detection Kit I (BD Biosciences, San Diego, CA, USA). After 72 h of treatment, INS‐1 cells were harvested. Annexin V‐FITC was added, and the cells were incubated on ice in the dark for 15 min. PI was then added, and cells were incubated for an additional 3 min on ice in the dark. Apoptotic cell numbers were analyzed using a FACSort flow cytometer (Becton, Dickinson and Company, Franklin Lakes, NJ, USA).

### 2.4 Western Blotting

Western blotting was performed to determine the levels of p‐c‐Abl, total c‐Abl, glutathione S‐transferase P1 (GSTP1), phosphorylated JNK (p‐JNK), phosphorylated p38 (p‐p38), and phosphorylated extracellular signal‐regulated kinase 1/2 (p‐ERK1/2). After 48 h of treatment, whole‐cell proteins were extracted from INS‐1 cells using radioimmunoprecipitation assay (RIPA) buffer (Sigma‐Aldrich, St. Louis, MO, USA) supplemented with Halt protease inhibitor cocktail (Thermo Fisher Scientific, Rockford, IL, USA). Protein concentrations were determined using a Micro BCA Protein Assay Kit (Thermo Fisher Scientific, Rockford, IL, USA) according to the manufacturer's instructions. Proteins were separated by gel electrophoresis and transferred to PVDF membranes (Bio‐Rad Laboratories, Hercules, CA, USA) using the Trans‐Blot Turbo Transfer System (Bio‐Rad Laboratories, Hercules, CA, USA). Membranes were then blocked with 5% non‐fat milk in Tris‐buffered saline with Tween‐20 (TBST) at room temperature for 1 h and incubated with primary antibodies diluted in 5% non‐fat milk in TBST at 4°C overnight. Primary antibodies were mouse monoclonal antibodies against p‐c‐Abl, total c‐Abl, GSTP1, p‐JNK, p‐p38, p‐ERK1/2, and β‐actin (all from Santa Cruz Biotechnology, Inc., Dallas, TX, USA). Membranes were probed with appropriate horseradish peroxidase‐conjugated secondary antibodies (Dako Denmark, Glostrup, Denmark) diluted in 5% non‐fat milk in TBST at room temperature for 1 h and bands were visualized by enhanced chemiluminescence (Thermo Fisher Scientific, Rockford, IL, USA) on X‐ray films. Quantification of protein band intensity was performed using ImageJ software (version 1.43; National Institutes of Health, Bethesda, MD, USA).

### 2.5 Nitro Blue Tetrazolium Chloride (NBT) Reduction Assay

Superoxide generation was assessed by NBT reduction assay. INS‐1 cells were seeded in triplicate in a clear 96‐well plate and, after approximately 24 h, were treated as described above. Following 48 h of treatment, NBT (1 mg/mL) (Sigma‐Aldrich, St. Louis, MO, USA) was added to each well, and cells were incubated for 90 min at room temperature. Cells were then lysed using 2 M potassium hydroxide (KOH) (Sigma‐Aldrich, St. Louis, MO, USA). The resulting formazan precipitates released from cells were dissolved in DMSO. Superoxide generation was quantified by measuring the optical density at 630 nm using a PowerWave microplate scanning spectrophotometer (BioTek Instruments, Inc., Winooski, VT, USA).

### 2.6 Caspase‐Glo 3/7 Assay

INS‐1 cells were seeded in triplicate in a white 96‐well plate with a white bottom and, after approximately 24 h, were treated as described above. The total volume per well was 100 µL. Cells were treated for 72 h. Three wells containing only culture medium without cells served as blank controls. To assess caspase‐3/7 activity, a Caspase‐Glo 3/7 assay (Promega, Madison, WI, USA) was performed according to the manufacturer's instructions with slight modifications. Briefly, 100 µL of Caspase‐Glo 3/7 reagent was added to each well. The plate was shaken at 300–500 rpm for 30 s and then incubated in the dark at 37°C for 30 min. Luminescence was measured using a BioTek Synergy H1 microplate reader (Agilent Technologies, Inc., Santa Clara, CA, USA). Caspase‐3/7 activity in each sample well was calculated by subtracting the average luminescence signal of the three blank control wells from the luminescence signal of that well.

### 2.7 Statistical Analysis

Data were analyzed using GraphPad Prism 5.01 software (GraphPad, San Diego, CA, USA) and presented as mean ± standard error of the mean (SEM). Statistically significant differences between groups were determined by one‐way analysis of variance (ANOVA) followed by Tukey's post hoc test. A *p* < 0.05 was considered statistically significant.

### 2.8 Use of Artificial Intelligence Generated Content (AIGC) Tools

OpenAI's ChatGPT at https://chat.openai.com was used to improve the language and readability of parts of this manuscript. Following the use of this tool, the authors reviewed and edited the content as appropriate and assume full responsibility for the content of the published article.

## 3 Results

### 3.1 Effects of Dexamethasone and Prunetin on Pancreatic β‐Cell Apoptosis

**Figure 1:**
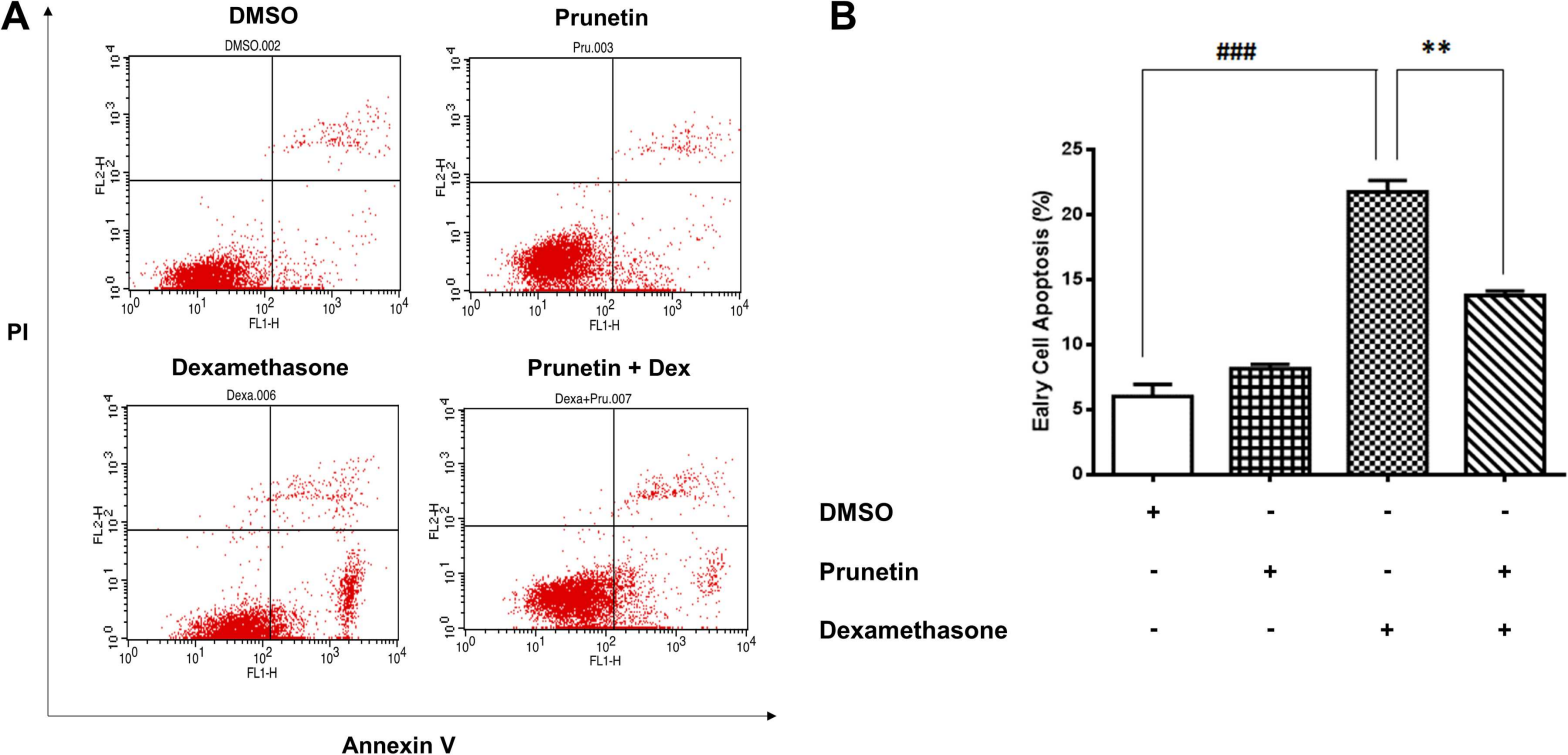
Prunetin attenuates dexamethasone‐induced pancreatic β‐cell apoptosis. INS‐1 cells were seeded and maintained for approximately 24 h before treatment with fresh medium containing the indicated agents. Four groups were established: (1) control (DMSO); (2) prunetin alone (10 µM, 1‐h pre‐treatment, maintained thereafter); (3) dexamethasone alone (0.1 µM); and (4) prunetin + dexamethasone (10 µM prunetin pre‐treatment for 1 h followed by 0.1 µM dexamethasone without prunetin removal). Cells were treated for 72 h, and apoptosis was assessed by an Annexin V‑fluorescein isothiocyanate/propidium iodide (Annexin V‐FITC/PI) assay. (A) Representative flow cytometry dot plots. (B) Early cell apoptosis percentage calculated from three independent experiments. Data are presented as mean ± SEM. ^###^ *p* < 0.001 versus DMSO control. **p < 0.01 versus dexamethasone alone.

To evaluate β‐cell apoptosis, an Annexin V‐FITC/PI assay was performed in INS‐1 cells. Dexamethasone treatment markedly increased the proportion of early apoptotic cells compared to the control (DMSO) (Figure 1A,B). Pre‐treatment with prunetin prior to dexamethasone exposure significantly reduced early apoptosis relative to dexamethasone alone (Figure 1A,B). These findings suggest that prunetin attenuates dexamethasone‐induced β‐cell apoptosis.

### 3.2 Effects of Dexamethasone and Prunetin on p‐c‐Abl, total c‐Abl, and GSTP1 Levels and Superoxide Generation in Β‐Cells

**Figure 2:**
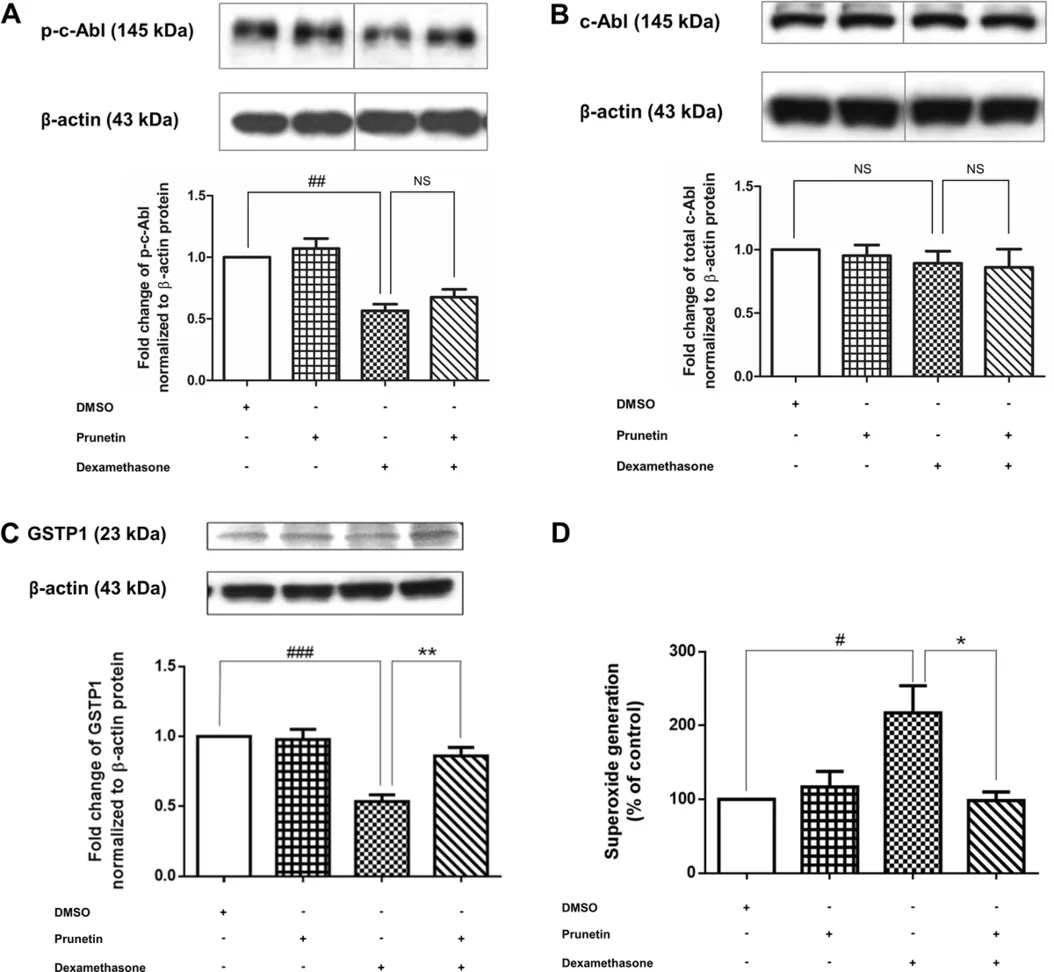
Prunetin does not restore dexamethasone‐suppressed p‐c‐Abl levels but counteracts dexamethasone‐induced GSTP1 downregulation and oxidative stress, whereas total c‐Abl levels remain unchanged in β‐cells. INS‐1 cells were seeded and maintained for approximately 24 h before treatment with fresh medium containing the indicated agents. Four groups were established: (1) control (DMSO); (2) prunetin alone (10 µM, 1‐h pre‐treatment, maintained thereafter); (3) dexamethasone alone (0.1 µM); and (4) prunetin + dexamethasone (10 µM prunetin pre‐treatment for 1 h followed by 0.1 µM dexamethasone without prunetin removal). Cells were treated for 48 h and analyzed by western blotting for p‐c‐Abl (phosphorylated cellular Abelson tyrosine kinase) (A), total c‐Abl (B), and GSTP1 (glutathione S‐transferase P1) (C) protein levels, or by NBT (nitro blue tetrazolium chloride) reduction assay for superoxide generation (D). (A, B) Representative western blots and quantification of p‐c‐Abl (A) and total c‐Abl (B), assessed separately and each normalized independently to β‐actin (fold change relative to control), from three independent experiments. For each protein, all displayed lanes were from the same gel and imaged under the same exposure conditions; vertical lines indicate omitted intervening lanes, resulting in non‐adjacent lanes in the displayed image. (C) A representative Western blot and quantification of GSTP1 levels normalized to β‐actin (fold change relative to control) from four independent experiments. (D) Superoxide generation quantified from three independent experiments and reported as % of control. Data are presented as mean ± SEM. ^#^ *p* < 0.05, ^##^ *p* < 0.01, ^###^ *p* < 0.001 versus DMSO control. *p < 0.05, **p < 0.01 versus dexamethasone alone. NS, not significant.

To determine the effects of dexamethasone and prunetin on the levels of p‐c‐Abl, total c‐Abl, and the detoxifying enzyme GSTP1, western blotting was performed. Dexamethasone significantly reduced p‐c‐Abl levels in INS‐1 cells compared to the control, and prunetin pre‐treatment did not significantly change p‐c‐Abl levels relative to dexamethasone alone (Figures 2A and S1). Total c‐Abl protein levels did not differ significantly among the four treatment groups (Figures 2B and S2). Dexamethasone significantly decreased GSTP1 levels compared to the control, whereas prunetin pre‐treatment before dexamethasone administration increased GSTP1 levels relative to dexamethasone alone (Figures 2C and S3). Superoxide generation was markedly increased by dexamethasone and was attenuated by prunetin pre‐treatment (Figure 2D). These findings indicate that prunetin does not restore dexamethasone‐suppressed p‐c‐Abl levels but mitigates the dexamethasone‐associated decrease in GSTP1 levels and increase in oxidative stress in β‐cells.

### 3.3 Effects of Dexamethasone and Prunetin on p‐JNK, p‐p38, and p‐ERK1/2 Levels in β‐Cells

**Figure 3:**
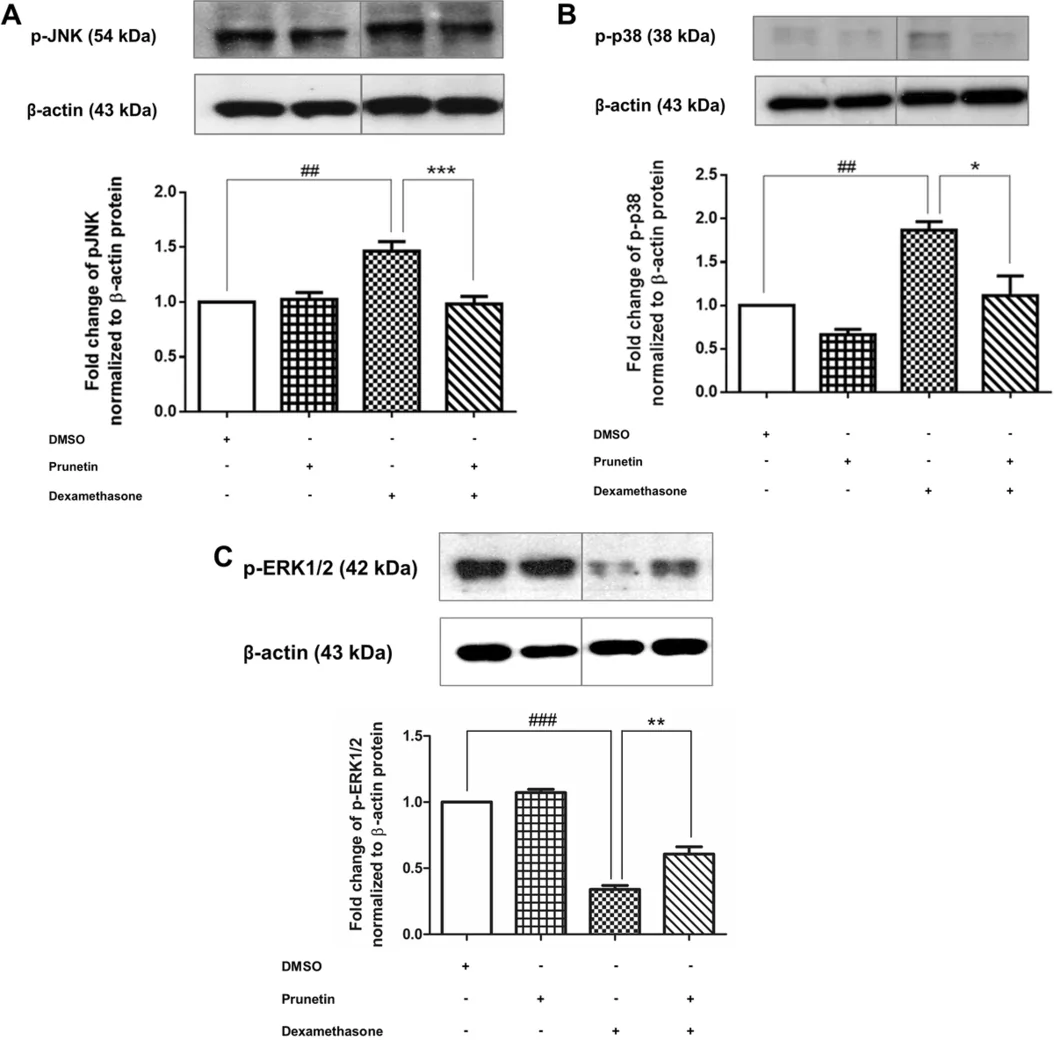
Prunetin attenuates dexamethasone‐induced alterations in p‐JNK, p‐p38, and p‐ERK1/2 levels in β‐cells. INS‐1 cells were seeded and maintained for approximately 24 h before treatment with fresh medium containing the indicated agents. Four groups were established: (1) control (DMSO); (2) prunetin alone (10 µM, 1‐h pre‐treatment, maintained thereafter); (3) dexamethasone alone (0.1 µM); and (4) prunetin + dexamethasone (10 µM prunetin pre‐treatment for 1 h followed by 0.1 µM dexamethasone without prunetin removal). Cells were treated for 48 h and analyzed by western blotting for p‐JNK (phosphorylated c‐Jun N‐terminal kinase) (A), p‐p38 (phosphorylated p38 mitogen‐activated protein kinase) (B), and p‐ERK1/2 (phosphorylated extracellular signal‐regulated kinase 1/2) (C) protein levels. For p‐JNK (A), a representative western blot and the corresponding quantification of target protein levels normalized to β‐actin (fold change relative to control) from four independent experiments are shown. For p‐p38 (B) and p‐ERK1/2 (C), data are derived from three independent experiments. For each protein, all displayed lanes were from the same gel and imaged under the same exposure conditions; vertical lines indicate omitted intervening lanes, resulting in non‐adjacent lanes in the displayed image. Data are presented as mean ± SEM. ^##^ *p* < 0.01, ^###^ *p* < 0.001 versus DMSO control. *p < 0.05, **p < 0.01, ***p < 0.001 versus dexamethasone alone.

Western blotting was performed to investigate the effects of dexamethasone and prunetin on p‐JNK, p‐p38, and p‐ERK1/2 levels in INS‐1 cells. Dexamethasone increased p‐JNK and p‐p38 levels but decreased p‐ERK1/2 levels compared to the control (Figures 3A–C and S4–S6). Pre‐treatment with prunetin before dexamethasone exposure reduced p‐JNK and p‐p38 levels and increased p‐ERK1/2 levels compared to dexamethasone alone (Figures 3A–C and S4–S6). These findings suggest that prunetin attenuates dexamethasone‐induced changes in p‐JNK, p‐p38, and p‐ERK1/2 levels in β‐cells.

### 3.4 Effects of Dexamethasone and Prunetin on Caspase‐3/7 Activity in β‐Cells

**Figure 4:**
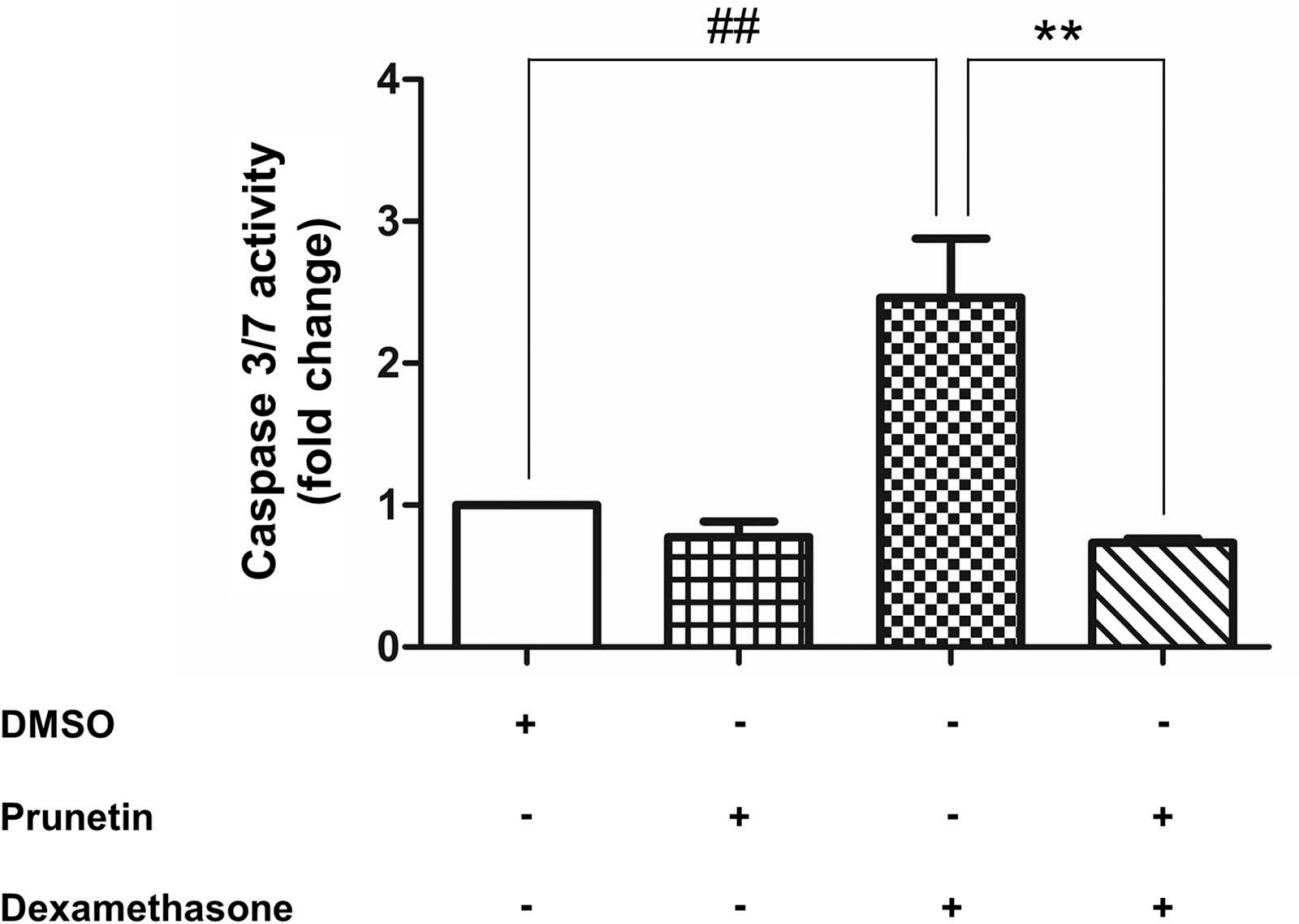
Prunetin decreases dexamethasone‐induced caspase‐3/7 activity in β‐cells. INS‐1 cells were seeded and maintained for approximately 24 h before treatment with fresh medium containing the indicated agents. Four groups were established: (1) control (DMSO); (2) prunetin alone (10 µM, 1‐h pre‐treatment, maintained thereafter); (3) dexamethasone alone (0.1 µM); and (4) prunetin + dexamethasone (10 µM prunetin pre‐treatment for 1 h followed by 0.1 µM dexamethasone without prunetin removal). Cells were treated for 72 h and subjected to a Caspase‐Glo 3/7 assay. Caspase‐3/7 activity (fold change relative to control) was calculated from three independent experiments. Data are presented as mean ± SEM. ^##^ *p* < 0.01 versus DMSO control. **p < 0.01 versus dexamethasone alone.

To investigate the effects of dexamethasone and prunetin on apoptotic pathways, caspase‐3/7 activity was determined in INS‐1 cells. Dexamethasone significantly increased caspase‐3/7 activity compared to the control, whereas prunetin pre‐treatment before dexamethasone administration reduced caspase‐3/7 activity relative to dexamethasone alone (Figure 4). These results suggest that prunetin attenuates dexamethasone‐induced caspase‐3/7 activity in β‐cells.

## 4 Discussion

Dexamethasone has been widely reported to induce β‐cell apoptosis [10, 11, 12, 13, 14], and the present findings are consistent with this effect. Pre‐treatment with prunetin significantly reduced dexamethasone‐induced apoptosis in INS‐1 cells. This is consistent with a previous report [11] and with work by Yang et al., which showed that prunetin improved survival in a mouse model of lipopolysaccharide (LPS)‐induced septic shock [28]. These data further support that prunetin attenuates dexamethasone‐induced β‐cell apoptosis. An earlier study suggested that suppression of p53 is an associated pathway [11]; the current work investigated whether additional pathways involving c‐Abl phosphorylation, oxidative stress, and MAPK signaling occurred concurrently with this protective effect of prunetin.

Dexamethasone increased superoxide production in INS‐1 cells, consistent with oxidative stress as a contributor to β‐cell apoptosis. Oxidative stress has been reported to activate c‐Abl, which promotes apoptosis and cell cycle arrest in various cell types [15, 16, 17, 18]. Evidence linking glucocorticoids to c‐Abl activation is limited, with a report in multiple myeloma cells [31]. No prior reports directly link dexamethasone to altered p‐c‐Abl levels in β‐cells. Unexpectedly, the results show that dexamethasone suppressed p‐c‐Abl levels despite increasing oxidative stress. The reduction in p‐c‐Abl levels, together with the absence of a detectable change in total c‐Abl abundance, is consistent with altered c‐Abl phosphorylation rather than altered overall c‐Abl protein expression. One possible explanation is that glucocorticoids interfere with upstream kinases or cofactors required for c‐Abl phosphorylation, thereby overriding oxidative stress–associated activation normally expected. Alternatively, β‐cells may possess mechanisms that uncouple oxidative stress from c‐Abl regulation. Pre‐treatment with prunetin did not significantly alter dexamethasone‐suppressed p‐c‐Abl levels, suggesting that modulation of c‐Abl phosphorylation may not be associated with prunetin‐mediated attenuation of β‐cell apoptosis.

GSTP1, a detoxifying enzyme that protects against oxidative stress and regulates JNK signaling [32], was subsequently examined. Dexamethasone reduced GSTP1 protein expression in INS‐1 cells, consistent with a previous study [10], potentially weakening antioxidant defenses and rendering β‐cells more vulnerable to oxidative stress. Similar observations have been reported in hepatocytes and lens epithelial cells, where GSTP1 downregulation enhanced reactive oxygen species accumulation and apoptosis [33, 34]. Prunetin restored GSTP1 expression and reduced superoxide production, suggesting that GSTP1 may be involved in the protective effects of prunetin.

MAPK signaling also contributes to dexamethasone‐induced apoptosis. Consistent with previous findings [10, 35], dexamethasone increased p‐JNK and p‐p38 levels, while reducing p‐ERK1/2 levels. These changes are consistent with stress‐induced apoptosis, as JNK and p38 promote cell death, whereas ERK1/2 supports cell survival [24, 36, 37, 38]. Prunetin attenuated the dexamethasone‐induced increase in p‐JNK and p‐p38 levels. These effects are consistent with a study in macrophages [28], where prunetin suppressed the LPS‐induced p‐JNK and p‐p38 levels. The present study also shows that prunetin attenuated the dexamethasone‐induced reduction of p‐ERK1/2 levels. Collectively, these findings suggest that prunetin‐mediated protection of β‐cells may be associated with rebalancing MAPK signaling toward survival.

Dexamethasone also increased caspase‐3/7 activity, consistent with apoptosis execution, whereas prunetin suppressed this activation. Taken together, the results indicate that dexamethasone‐induced β‐cell apoptosis occurs concurrently with altered redox status, reduced GSTP1 expression, altered MAPK signaling, and reduced p‐c‐Abl levels, while total c‐Abl abundance remains unchanged. In contrast, prunetin pre‐treatment is linked to restoration of GSTP1 expression, reduction of oxidative stress, and modulation of MAPK signaling, but not to restoration of dexamethasone‐suppressed p‐c‐Abl levels. Overall, this study provides insight into pathways potentially involved in prunetin‐mediated attenuation of dexamethasone‐induced β‐cell apoptosis. Further studies in primary pancreatic islets or *in vivo* models may extend these observations to more complex physiological systems. Additionally, mechanistic investigations using pharmacological or genetic approaches could further elucidate the relationships among the signaling pathways examined.

## 5 Conclusions

Prunetin attenuates dexamethasone‐induced pancreatic β‐cell apoptosis, and this effect is associated with modulation of oxidative stress, GSTP1 expression, and MAPK signaling pathways, but not with restoration of dexamethasone‐suppressed p‐c‐Abl levels. These findings suggest the potential relevance of prunetin in the context of glucocorticoid‐induced β‐cell injury, a process implicated in steroid‐induced diabetes, and highlight GSTP1 and MAPK pathways as candidates for further mechanistic investigation.

## Author Contributions

Suwattanee Kooptiwut conceived and supervised the study and acquired funding. Thaksaon Kittipassorn and Suwattanee Kooptiwut designed the study. Thaksaon Kittipassorn, Nawarat Rattanajearakul, Petcharee Maneethorn, and Namoiy Semprasert conducted the experimental work and data acquisition procedures. All authors contributed to the analysis and interpretation of the experimental findings. Thaksaon Kittipassorn and Suwattanee Kooptiwut undertook the critical evaluation of the results, comparing them with existing conclusions, established knowledge and relevant theoretical frameworks, summarizing the findings and providing recommendations. Thaksaon Kittipassorn prepared the initial manuscript draft, and Thaksaon Kittipassorn and Suwattanee Kooptiwut reviewed and critically revised the manuscript. All authors reviewed and approved the final version of the manuscript.

## Conflicts of Interest

The authors declare no conflicts of interest.

## Supporting information


Supporting File

## Data Availability

The data that support the findings of this study are available from the corresponding author upon reasonable request.
