# Supplementary material for: Prunetin Attenuates Dexamethasone‐Induced Pancreatic β‐Cell Apoptosis: Modulation of Oxidative Stress and MAPK Signaling but Not Phosphorylated C‐Abl Levels
Source: Cell Biochem Funct. 2026 Aug 24;44(8):e70292. doi: 10.1002/cbf.70292 (PMC13501189; doi:10.1002/cbf.70292)
Supplement: Supplementary file 1 [file CBF-44-e70292-s001.pdf]

Supplementary materials

Prunetin attenuates dexamethasone-induced pancreatic β-cell apoptosis: Modulation of oxidative stress and MAPK signaling but not phosphorylated c-Abl levels

Authors: Thaksaon Kittipassorn[a], Nawarat Rattanajearakul[a,1], Petcharee Maneethorn[a], Namoiy Semprasert[a], Suwattanee Kooptiwut[a,*]

Affiliation: [a]Department of Physiology, Faculty of Medicine Siriraj Hospital, Mahidol University, Bangkok 10700, Thailand

*Corresponding author. Assoc. Prof. Suwattanee Kooptiwut

Department of Physiology, Faculty of Medicine Siriraj Hospital,

Mahidol University, Bangkok 10700, Thailand

Tel: +662 419-5277

Email address: s_kooptiwut@hotmail.com, suwattanee.koo@mahidol.ac.th

[1]Other affiliation/present address: Laboratory of Alimentary Neuroscience, Department of Applied Life Sciences, Graduate School of Bioagricultural Sciences, Nagoya University, Nagoya, Aichi 464-8601, Japan

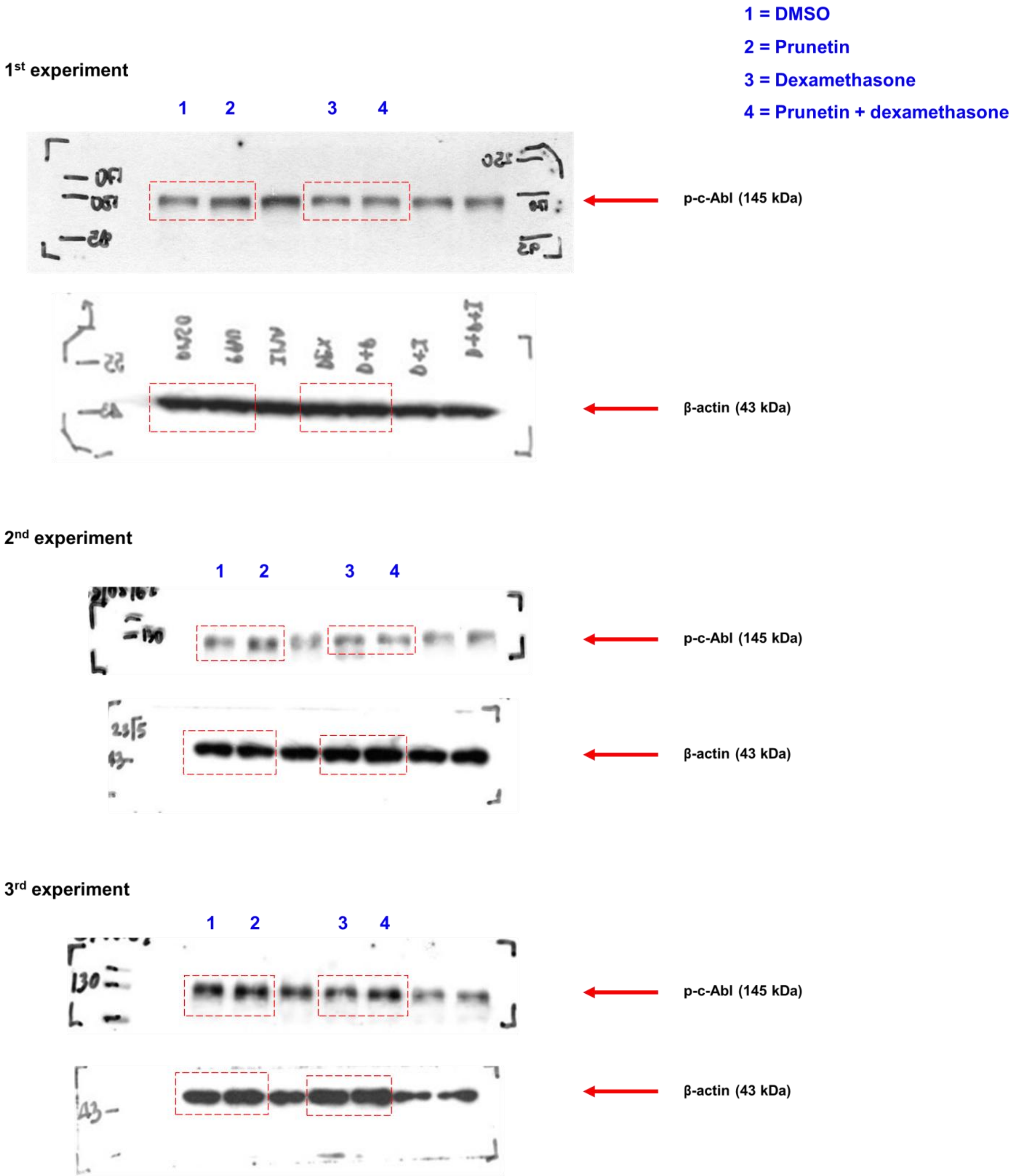

Supplementary Figure S1 (related to Figure 2A): Effects of dexamethasone and prunetin on p-c-Abl protein levels in β-cells. INS-1 cells were seeded and maintained for approximately 24 hours before treatment with fresh medium containing the indicated agents. Four groups were established: (1) control (DMSO); (2) prunetin alone (10 µM, 1-hour pre-treatment, maintained thereafter); (3) dexamethasone alone (0.1 µM); and (4) prunetin + dexamethasone (10 µM prunetin pre-treatment for 1 hour followed by 0.1 µM dexamethasone without prunetin removal). Cells were treated for 48 hours and analyzed by Western blotting for p-c-Abl protein levels, with β-actin as the loading control. Blots from three independent experiments are shown. Red dashed boxes indicate the protein bands used for quantification.

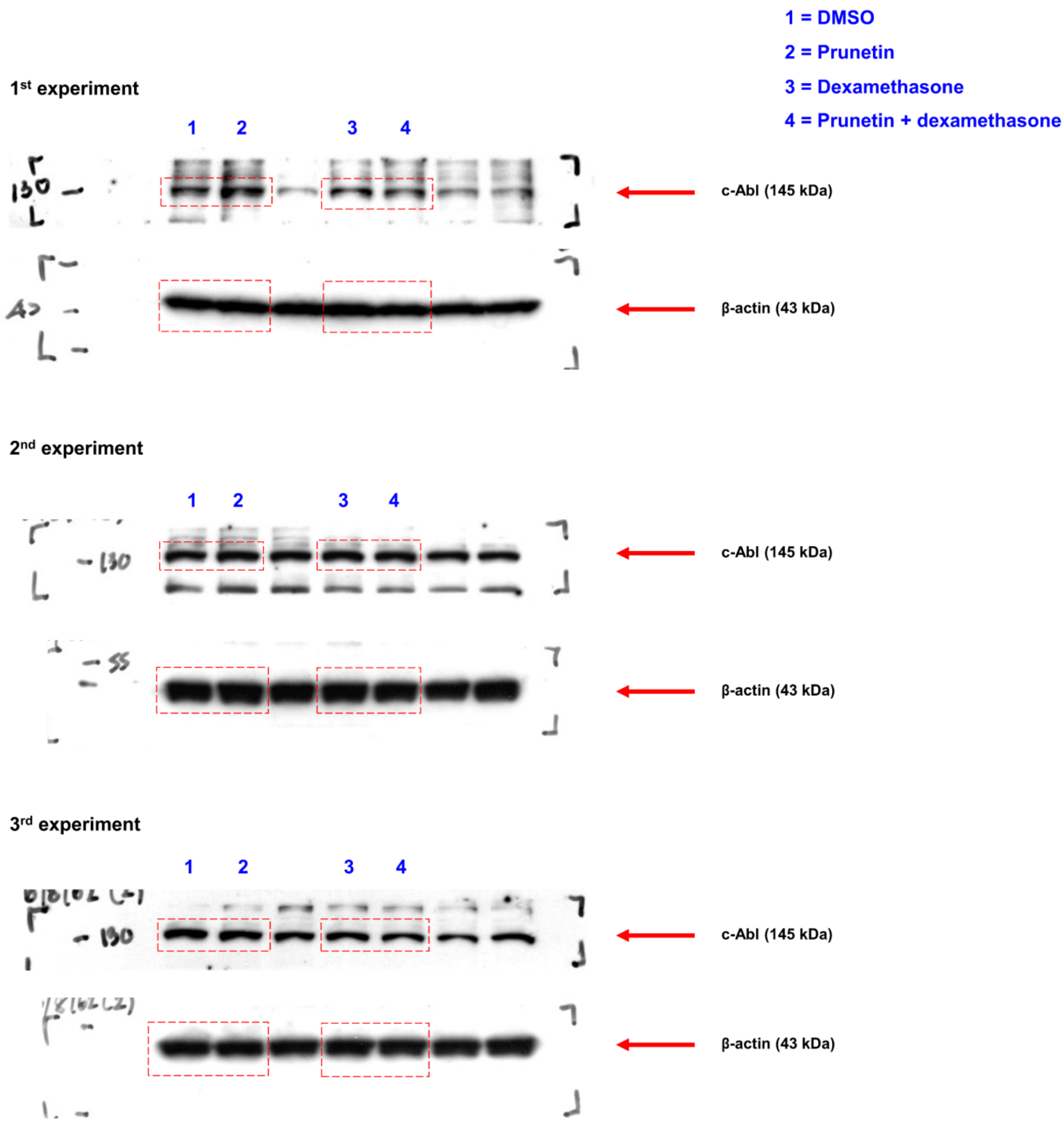

Supplementary Figure S2 (related to Figure 2B): Effects of dexamethasone and prunetin on total c-Abl protein levels in β-cells. INS-1 cells were seeded and maintained for approximately 24 hours before treatment with fresh medium containing the indicated agents. Four groups were established: (1) control (DMSO); (2) prunetin alone (10 µM, 1-hour pre-treatment, maintained thereafter); (3) dexamethasone alone (0.1 µM); and (4) prunetin + dexamethasone (10 µM prunetin pre-treatment for 1 hour followed by 0.1 µM dexamethasone without prunetin removal). Cells were treated for 48 hours and analyzed by Western blotting for total c-Abl protein levels, with β-actin as the loading control. Blots from three independent experiments are shown. Red dashed boxes indicate the protein bands used for quantification.

1 = DMSO

2 = Prunetin

3 = Dexamethasone

4 = Prunetin + dexamethasone

1st experiment

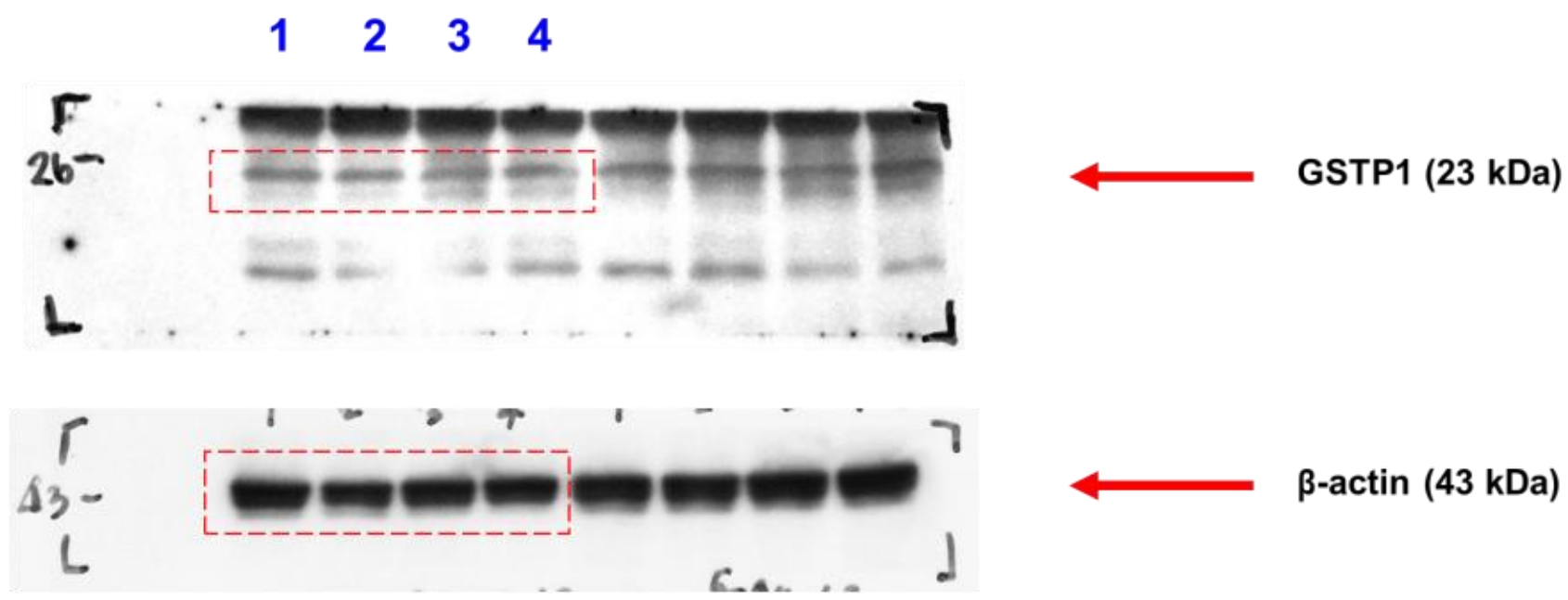

2nd experiment

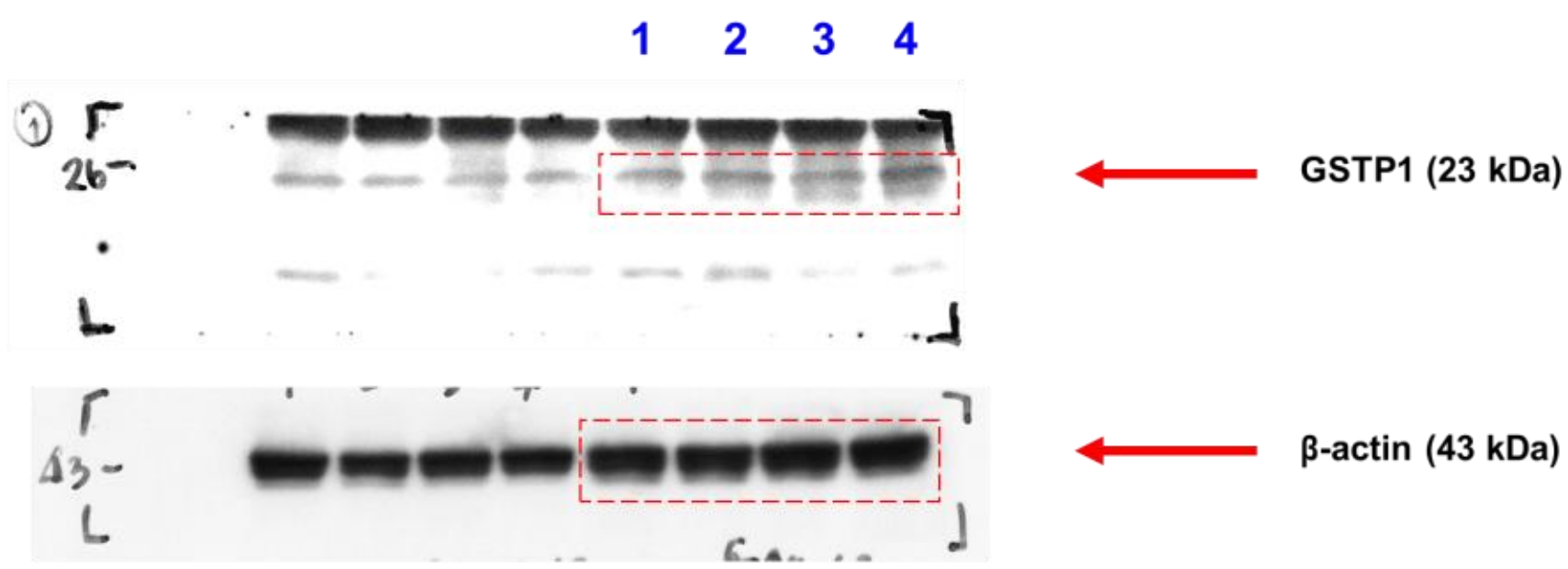

3rd experiment

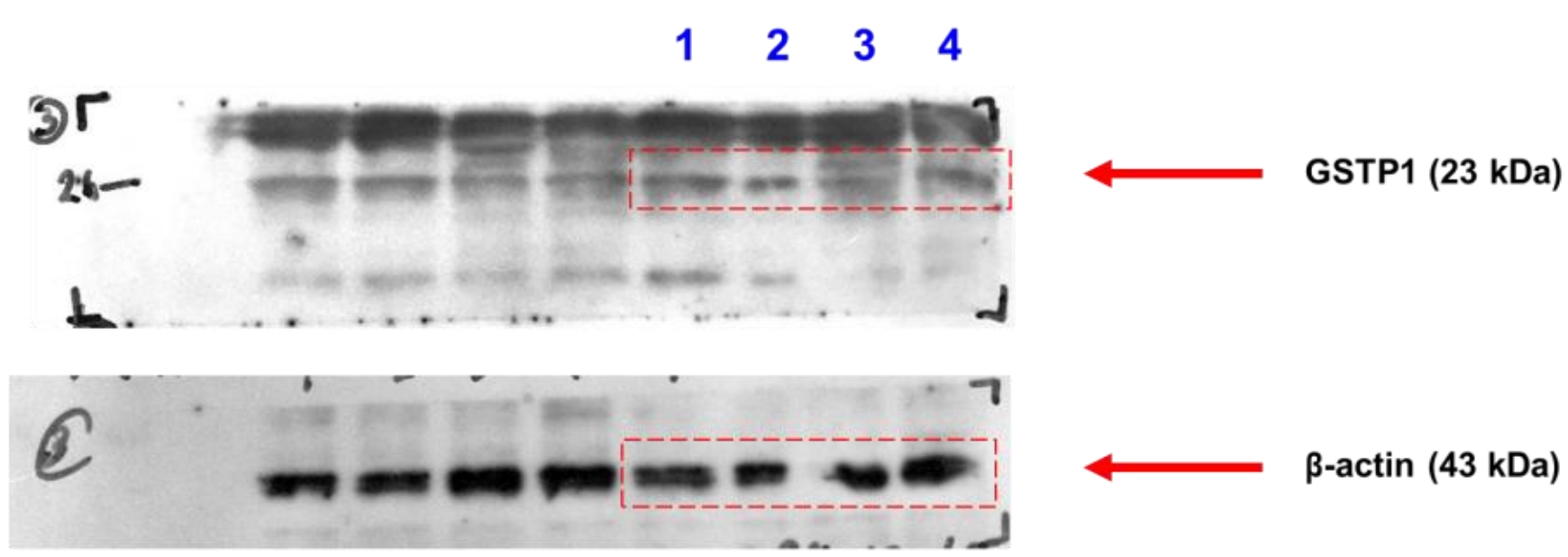

4th experiment

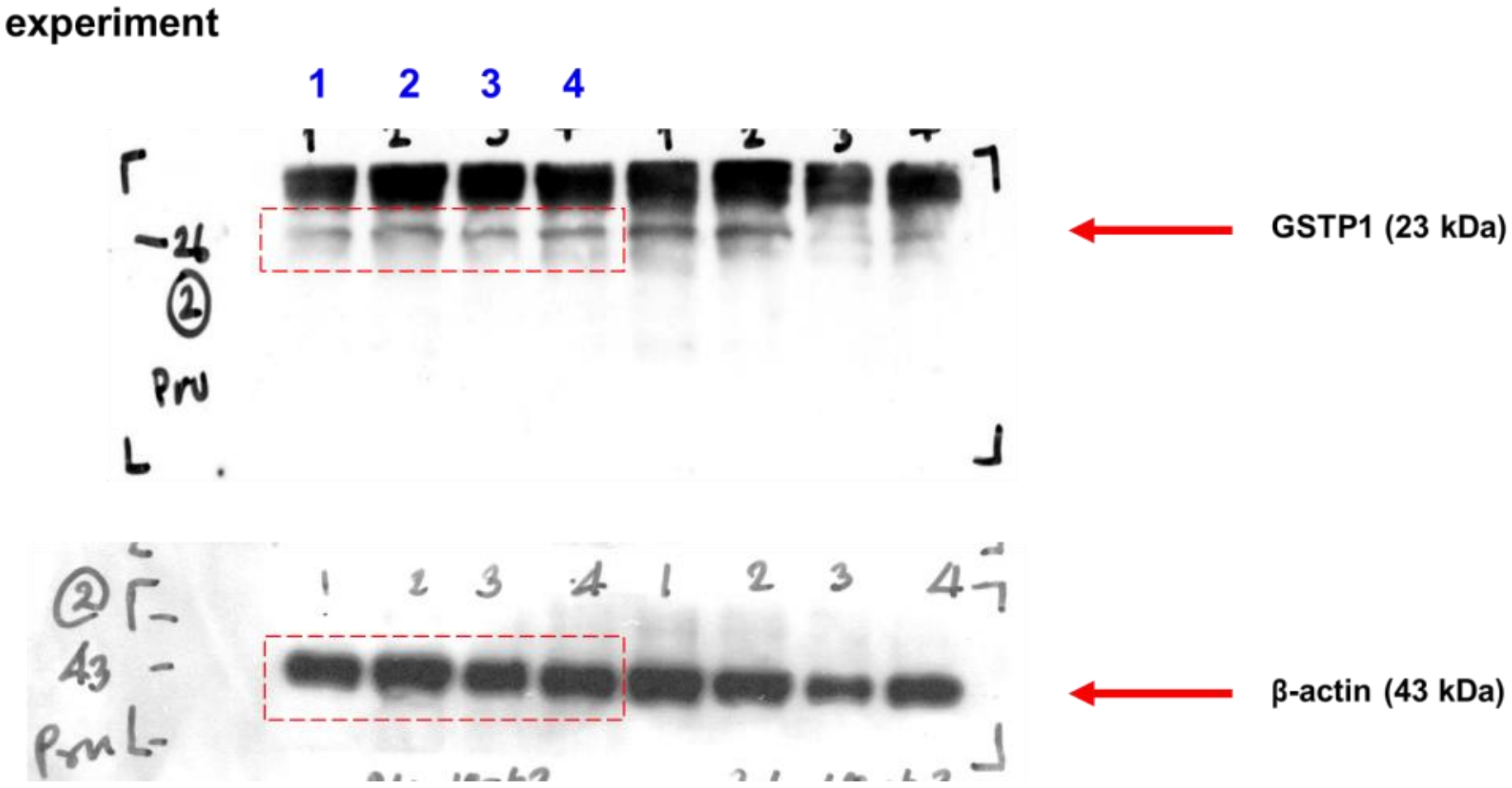

Supplementary Figure S3 (related to Figure 2C): Effects of dexamethasone and prunetin on GSTP1 protein levels in β-cells. INS-1 cells were seeded and maintained for approximately 24 hours before treatment with fresh medium containing the indicated agents. Four groups were established: (1) control (DMSO); (2) prunetin alone (10 µM, 1-hour pre-treatment, maintained thereafter); (3) dexamethasone alone (0.1 µM); and (4) prunetin + dexamethasone (10 µM prunetin pre-treatment for 1 hour followed by 0.1 µM dexamethasone without prunetin removal). Cells were treated for 48 hours and analyzed by Western blotting for GSTP1 protein levels, with β-actin as the loading control. Blots from four independent experiments are shown. Red dashed boxes indicate the protein bands used for quantification.

1 = DMSO

2 = Prunetin

3 = Dexamethasone

4 = Prunetin + dexamethasone

1st experiment

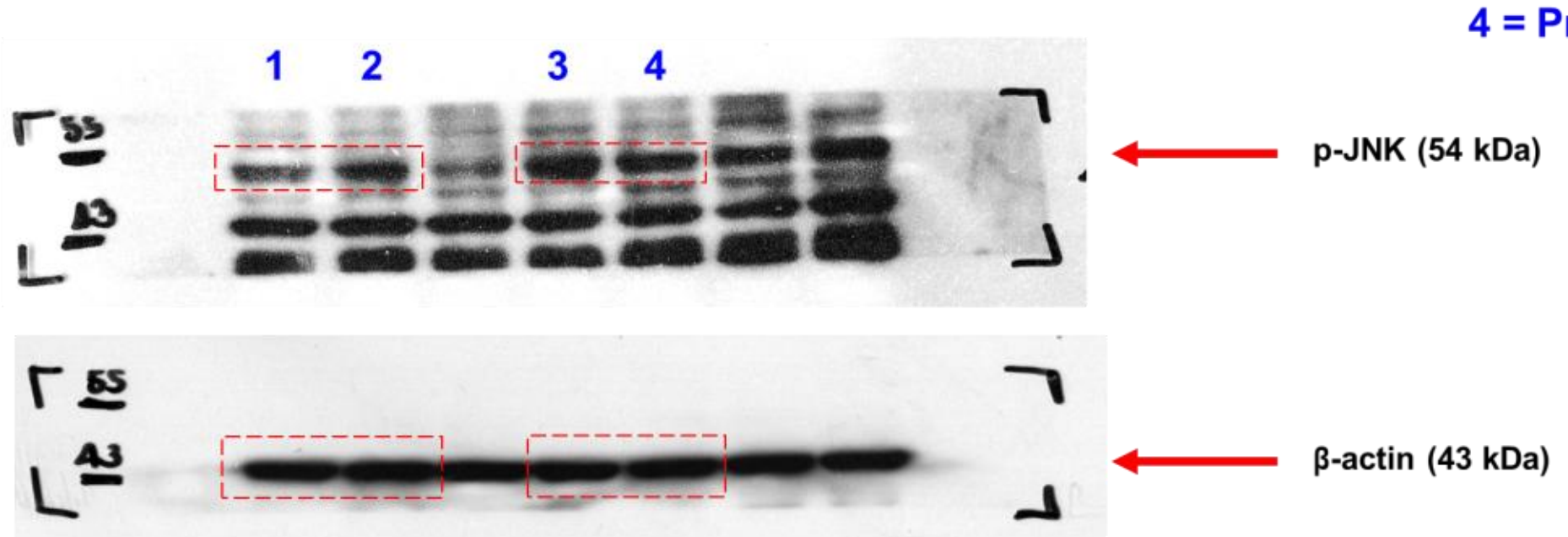

2nd experiment

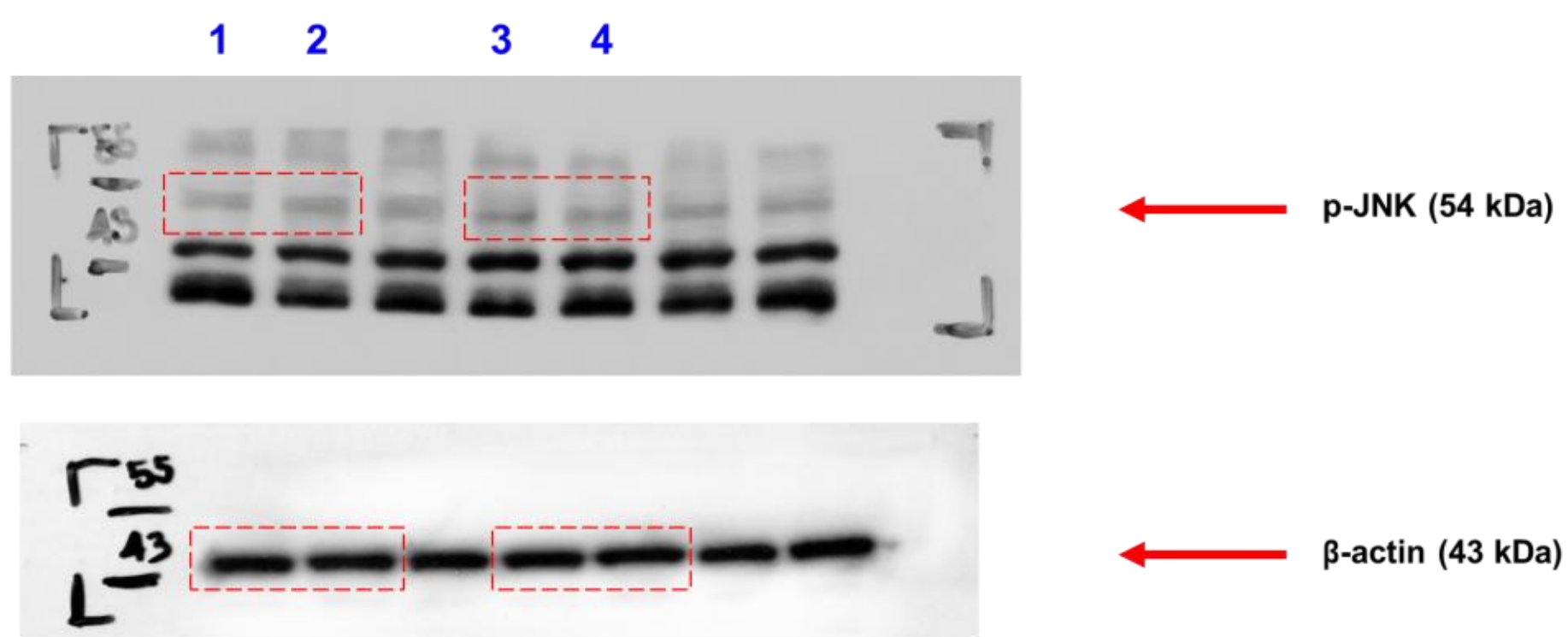

3rd experiment

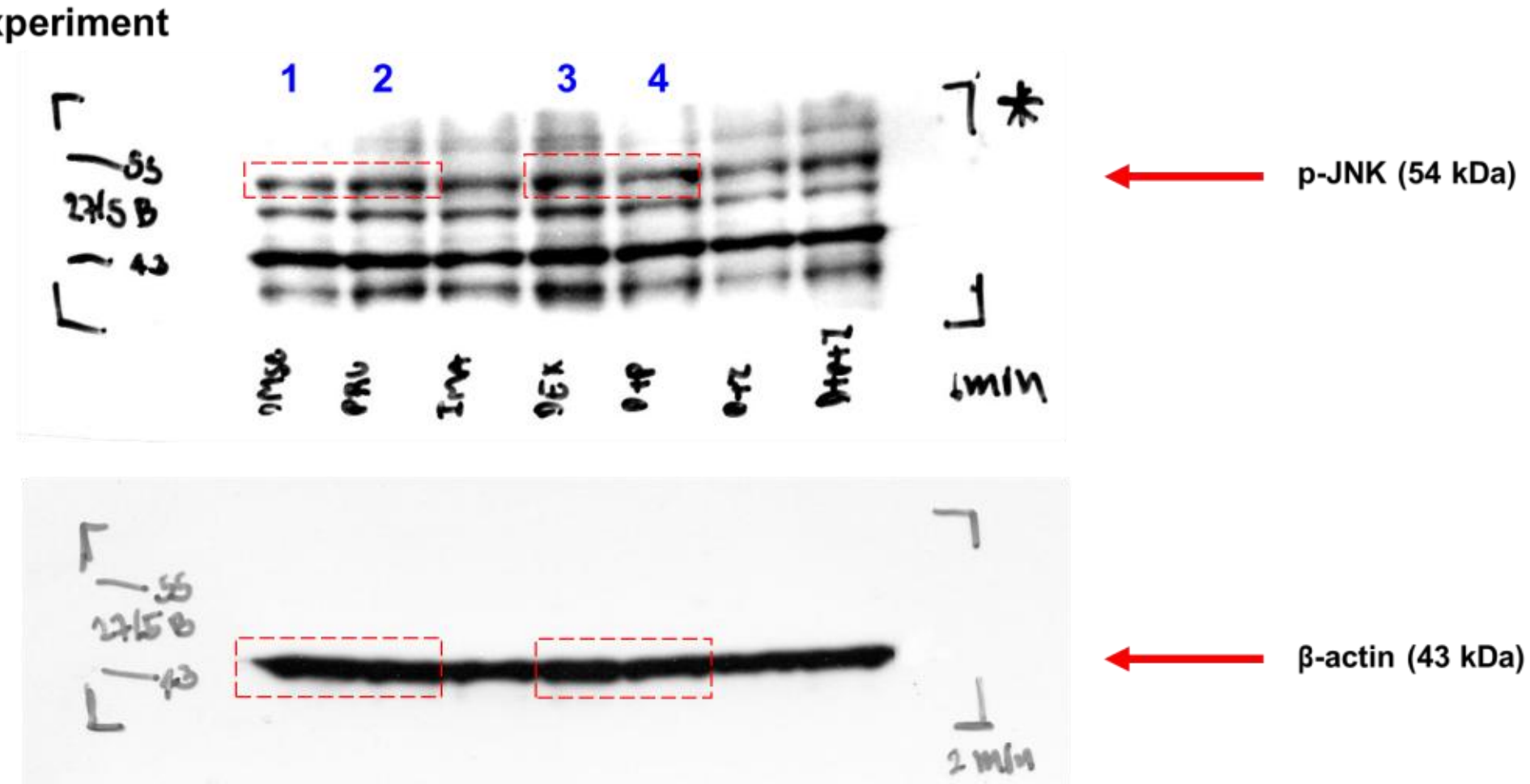

4th experiment

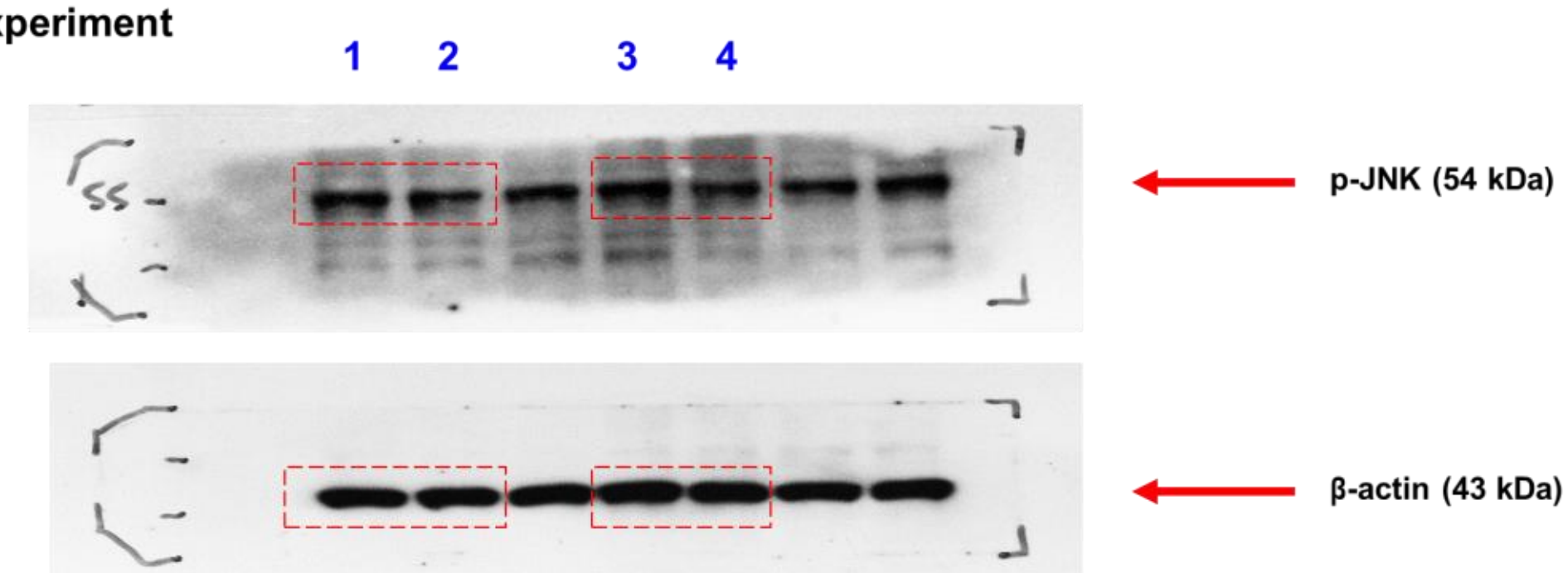

Supplementary Figure S4 (related to Figure 3A): Effects of dexamethasone and prunetin on p-JNK protein levels in β-cells. INS-1 cells were seeded and maintained for approximately 24 hours before treatment with fresh medium containing the indicated agents. Four groups were established: (1) control (DMSO); (2) prunetin alone (10 μM, 1-hour pre-treatment, maintained thereafter); (3) dexamethasone alone (0.1 μM); and (4) prunetin + dexamethasone (10 μM prunetin pre-treatment for 1 hour followed by 0.1 μM dexamethasone without prunetin removal). Cells were treated for 48 hours and analyzed by Western blotting for p-JNK protein levels, with β-actin as the loading control. Blots from four independent experiments are shown. Red dashed boxes indicate the protein bands used for quantification.

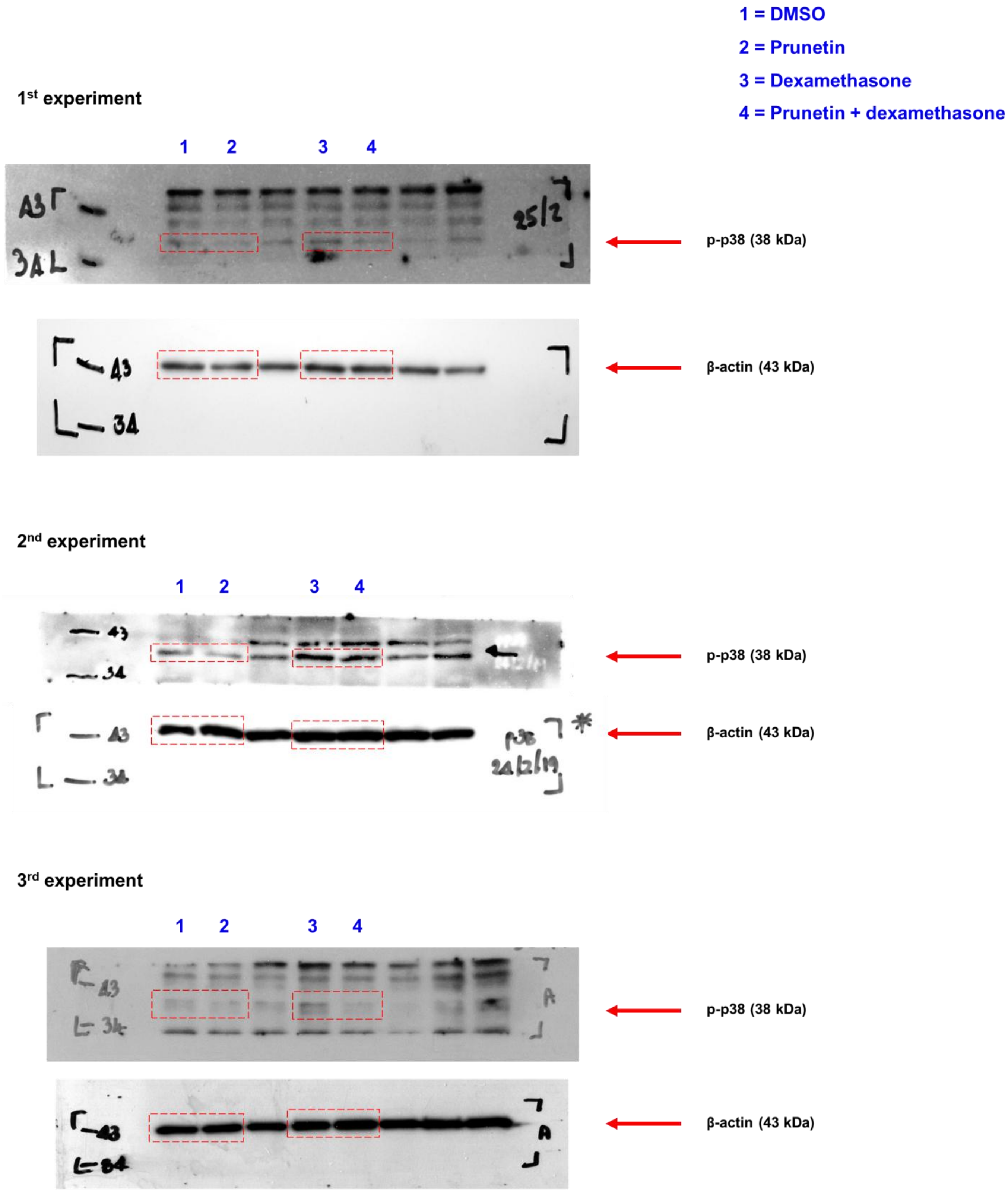

Supplementary Figure S5 (related to Figure 3B): Effects of dexamethasone and prunetin on p-p38 protein levels in β-cells. INS-1 cells were seeded and maintained for approximately 24 hours before treatment with fresh medium containing the indicated agents. Four groups were established: (1) control (DMSO); (2) prunetin alone (10 μM, 1-hour pre-treatment, maintained thereafter); (3) dexamethasone alone (0.1 μM); and (4) prunetin + dexamethasone (10 μM prunetin pre-treatment for 1 hour followed by 0.1 μM dexamethasone without prunetin removal). Cells were treated for 48 hours and analyzed by Western blotting for p-p38 protein levels, with β-actin as the loading control. Blots from three independent experiments are shown. Red dashed boxes indicate the protein bands used for quantification.

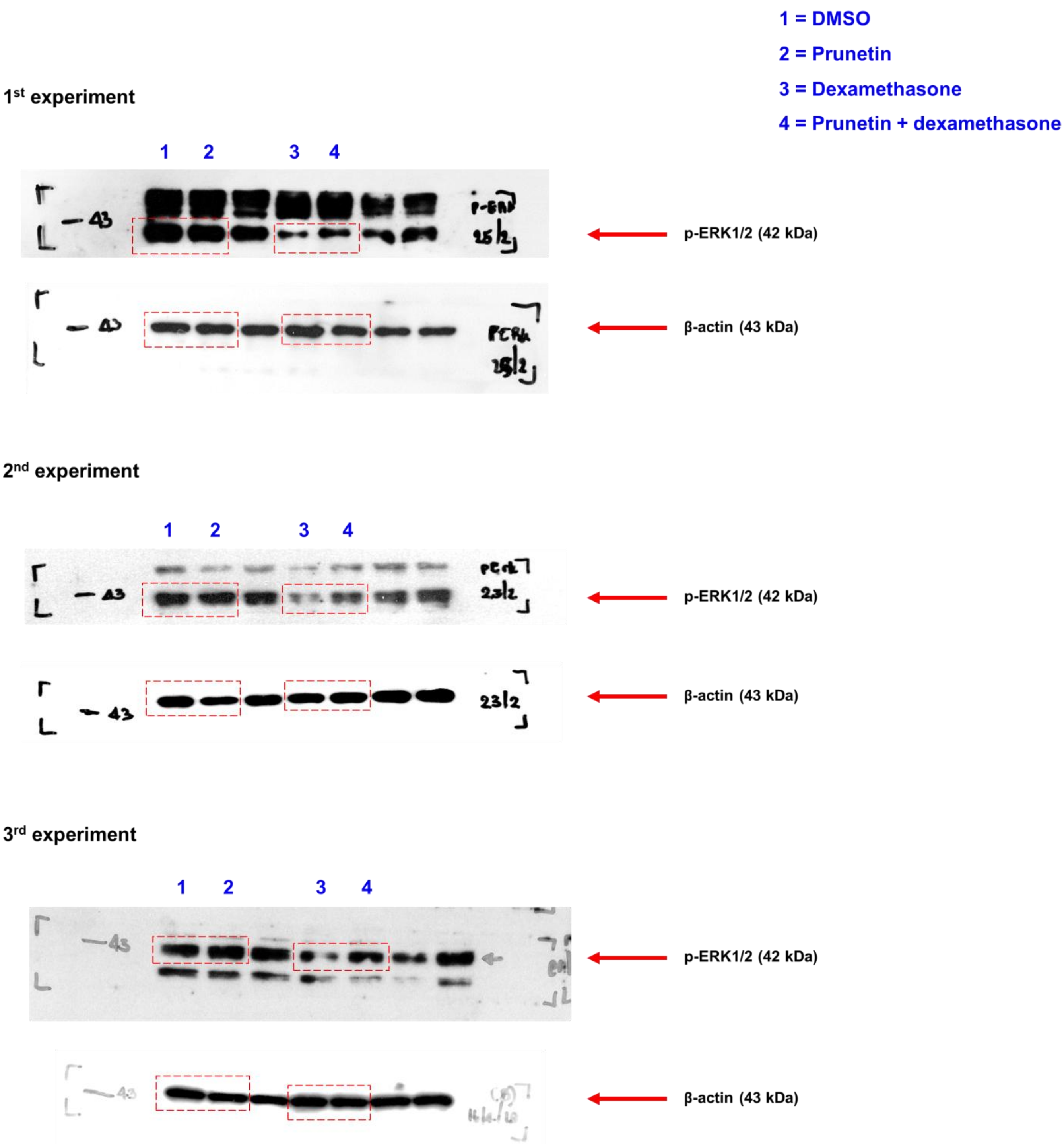

Supplementary Figure S6 (related to Figure 3C): Effects of dexamethasone and prunetin on p-ERK1/2 protein levels in β-cells. INS-1 cells were seeded and maintained for approximately 24 hours before treatment with fresh medium containing the indicated agents. Four groups were established: (1) control (DMSO); (2) prunetin alone (10 µM, 1-hour pre-treatment, maintained thereafter); (3) dexamethasone alone (0.1 µM); and (4) prunetin + dexamethasone (10 µM prunetin pre-treatment for 1 hour followed by 0.1 µM dexamethasone without prunetin removal). Cells were treated for 48 hours and analyzed by Western blotting for p-ERK1/2 protein levels, with β-actin as the loading control. Blots from three independent experiments are shown. Red dashed boxes indicate the protein bands used for quantification.
